# Digit Ratio and Dental Caries: A Sexually Dimorphic Trait

**DOI:** 10.5005/jp-journals-10005-1474

**Published:** 2017-02-01

**Authors:** Priya Verma, Amitha M Hegde

**Affiliations:** 1 Professor, Department of Pedodontics and Preventive Dentistry, K.D. Dental College & Hospital, Mathura, Uttar Pradesh, India; 2 Professor and Head, Department of Pedodontics and Preventive Dentistry, A.B. Shetty Memorial Institute of Dental Sciences, Mangaluru Karnataka, India

**Keywords:** Dental caries, Digit ratio, Hormonal fingerprint.

## Abstract

Dental caries is the most common oral health disease affecting all age groups, races, and geographic locations. The need for the study was to determine the anatomical marker that could predict the taste perception and caries at an early stage. Aim of the study was to determine the correlation between digit ratio and caries experience in school-going children of south Canara region. An observational and cross-sectional pattern was adopted for the present study. The study was then evaluated to find out the correlation between the digit ratio that is thought to be predetermined with caries experience in children of age group 6 to 16 years. In the total sample of 2,037 children, the total population was divided into two categories, i.e., high digit ratio and low digit ratio. Of the total population, 1,112 had low digit ratio and 925 had high digit ratio. Caries experience was highest in low-risk group, followed by moderate, high risk, low risk, and very high risk groups. In all the categories, low digit ratio was affected more than high digit ratio. The study clearly states a positive correlation between digit ratio, taste, social behavior, and dental caries.

**How to cite this article:** Verma P, Hegde AM. Digit Ratio and Dental Caries: A Sexually Dimorphic Trait. Int J Clin Pediatr Dent 2018;11(1):1-6.

## INTRODUCTION

The relative length of fingers on the human hand has attracted considerable research interest and has been linked to various psychological traits and diseases. The ratio of lengths of second to fourth digits (2D:4D) is a sexually dimorphic trait that is lower in males than in females. Researchers found that the second (index) finger is usually shorter than the fourth (ring) finger. George^1^ noticed that men’s ring finger was relatively longer than the index finger, i.e., low digit ratio, while in females it was more likely to show the opposite pattern, i.e., high digit ratio.

This sex difference was found to be existing in 2-year-old children that may have been established prenatally by the 13th or 14th week postconception and could be used as a stable parameter. Individuals who have shorter index finger are testosterone-derived individuals and those having longer index finger are estrogen-derived individuals.^2^

It is a common belief that living organism never operates in an open loop mechanism, i.e., whenever there is a stimulus there will be a response followed by a regulation. In a very similar way, hormones are stimulated that leads to a response, followed by their regulation, thereby bringing permanent changes in our body.

Literature also indicates that the fetus during intra-uterine life is exposed to prenatal androgens, and it is their concentration of exposure which dictates their taste perception, thereby affecting their oral health.^1^

### Aim

The aim of the study was to find out the existence of sexual dimorphism and if any association exists between digit ratio and oral health.

### Objectives

(i) To determine the existence of sexual dimorphism; (ii) to evaluate the stability and reproducibility of digit ratio; and (iii) to evaluate if digit ratio is associated with dental caries.

## MATERIALS AND METHODS

**Fig. 1:**
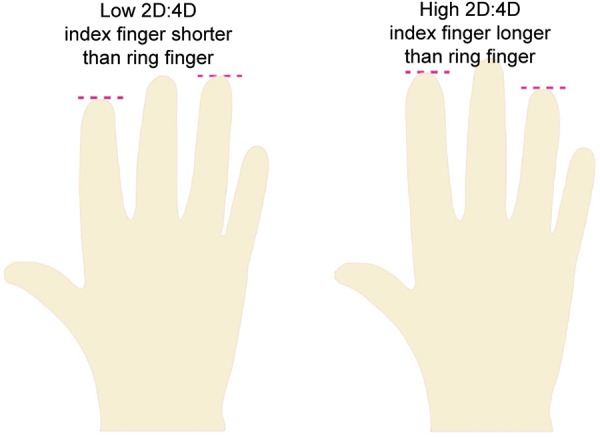
Diagrammatic representation of classification of digit ratio

A total number of 2,037 school-going children belonging to the age group of 6 to 16 years of both sexes that reported to the Department of Pedodontics and Preventive Children Dentistry, A.B. Shetty Memorial Institute of Dental Sciences, Deralakatte, Mangaluru, India, were a part of this study. The digit print was then recorded on right hand^3,4^ by measuring the length ratio of the index finger as well as the ring finger with the help of digital Vernier caliper and was divided into two categories, i.e., low digit ratio and high digit ratio. The caries experience [decay-missing-filled teeth (DMFT)/decayed-filled tooth (dft) index] was recorded using visible light, mouth mirror, and Community Periodontal Index probe. Caries risk assessment classification was done by the World Health Organization (WHO)^5^ oral health survey: basic methods. The children belonging to American Society of Anesthesiologist (ASA) 1 category was included in the study, while the children belonging to ASA group 2 to 5, children with special needs, hormonal imbalance, and children with distorted digits were excluded from the study (Fig. 1 and Table 1).

**Table 1:** **Table 1:** Caries risk assessment classification by the WHO

*Risk category*		*DMFT/dft*	
Very low		<1.2	
Low		1.2-2.6	
Moderate		2.7-4.4	
High		4.5-6.5	
Very high		>6.5	

## RESULTS AND OBSERVATIONS

To find out the existence of sexual dimorphism in the present study.

**Graph 1:**
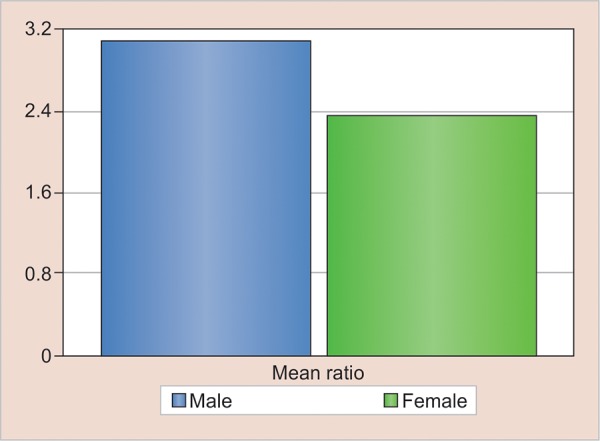
The mean ratio of male and female population

The 2D:4D ratio (2D/4D) of right hand was calculated for each individual and the tabulation was done according to the sex. Individual mean was calculated for the second and fourth digit, and the mean ratio of 2D:4D was taken out for both the sexes. The mean ratio in men (0.92) is found to be lower than in females (0.97), thereby suggesting the ratio between the two digits is smaller in males as compared with females and the existence of sexual dimorphism (Graph 1 and Table 2).

To observe the frequency of standardized 2D:4D digit ratio according to sex in a given population:

The standardized data were tabulated for the frequency of digit ratio according to sex, and it was observed with that of the total. Male population was 1,024, and the total female population was 1,013.

**Graph 2:**
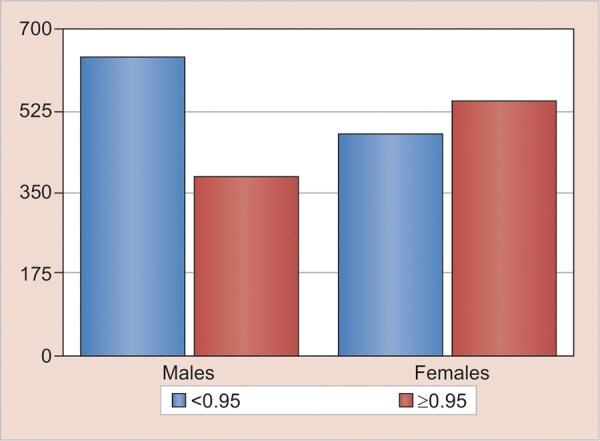
The frequency of digit ratio according to sex

The digit ratio was divided into two categories, i.e., low digit ratio category was 1,112 and high digit ratio category was 925. Male distribution was found to be 58% and female distribution was 42% in low digit ratio category, wherein in high digit ratio category female distribution was found to be 58% and male distribution was 42% (Graph 2 and Table 3).

**Graph 3:**
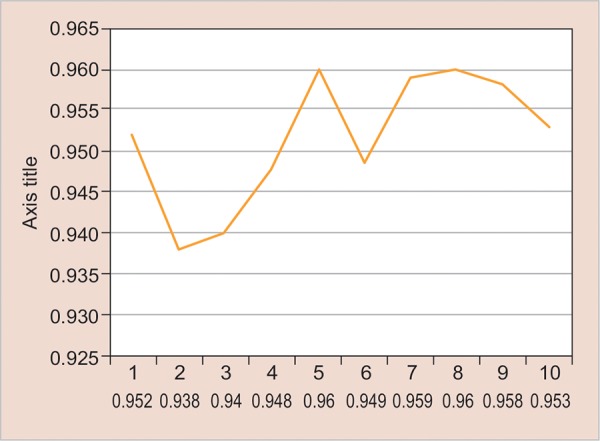
The frequency of mean digit ratio at different age intervals

To evaluate the stability and reproducibility of the digit ratio:

The data show the mean ratio between the two digits of the children between 6 and 16 years of the age. The minimum ratio seen was around 0.94 and the maximum was 0.96. The mean ratio was around 0.95. No significant changes were seen when all the age groups were compared. The above-mentioned observation is suggestive of the finding that digit ratio once established remains constant throughout the life and can be used as a stable parameter (Graph 3 and Table 4).

**Table 2:** **Table 2:** The mean ratio of male and female population

		*4D:2D*													
*Sex*		*Mean difference in length*		*SD*		*Std. error of mean*		*t-test value*		*p-value*		*Mean length of digits*		*Mean ratio of 2D:4D*	
Male		4.78		2.52		0.08		6.103		0.000***		2D: 54.01 4D: 58.90		0.92	
Female		1.98		2.86		0.09						2D: 54.94 4D: 56.96		0.97	

Independent samples test; ***Very highly significant difference (p-value ≤ 0.001)

**Table 3:** **Table 3:** The frequency of digit ratio according to sex

*Digit ratio*		*Male*		*Female*		*Mean*		*SD*		*t-test*	
<0.95 (1112)		641 (58%)		471 (42%)		0.92		0.027		41.648	
≥0.95 (925)		383 (42)		542 (58%)		0.98		0.044			
		1024 (100%)		1,013 (100%)				χ^2^ = 53.262		p < 0.001 VHS	

SD: Standard deviation; VHS: Very highly significant

**Table 4:** **Table 4:** The frequency of mean digit ratio at different age intervals

*Descriptives*											
*Age*		*n*		*Mean*		*Std. deviation*		*Minimum*		*Maximum*	
6		224		0.9517		0.04984		0.77		1.15	
7		210		0.9378		0.05510		0.57		1.13	
8		143		0.9317		0.04862		0.63		1.04	
9		226		0.9477		0.06564		0.85		1.45	
10		236		0.9622		0.31183		0.51		5.64	
11		157		0.9487		0.05238		0.75		1.21	
12		157		0.9591		0.05181		0.85		1.40	
13		320		0.9630		0.03955		0.83		1.09	
14		366		0.9583		0.04754		0.52		1.15	
		2039		0.9529		0.11660		0.51		5.64	

F = 1.7633; p = 0.08, nonsignificant

To evaluate the association of digit ratio with dental caries:

The caries experience in both the dentitions was observed as 49% in very low-risk category, followed by moderate risk (16.6%), high risk (12%), low risk (12%), and very high risk (10%).

Very low-risk group constituted 997 (49%) of total population. The group was further divided according to sex and digit ratio. The distribution of digit ratio was found to be 497 (49.8%) for low digit ratio and 500 (50.2%) for high digit ratio. The high digit ratio category had 52% of females and 48% of males. The low digit ratio category had 58% of males and 42% of females. The group was found to be very highly significant.

The low-risk group that formed around 249 (12%) of total population was further divided according to sex and digit ratio. The distribution of digit ratio was found to be 136 (55%) for low digit ratio and 113 (45%) for high digit ratio. The high digit ratio category had 56% of female and 44% of males. The low digit ratio category had 57% of males and 43% of females. The group was found to be very highly significant.

The moderate-risk group that formed around 339 (16.7%) of total population was further divided according to sex and digit ratio. The distribution of digit ratio was found to be 226 (66.6%) for low digit ratio and 113 (33.4%) for high digit ratio. The low digit ratio category had 55% of males and 45% of females. The high digit ratio category had 47% of males and 53% of females. The group was found to be very highly significant.

The high-risk group that constituted around 240 (12%) of total population was further divided according to sex and digit ratio. The distribution of digit ratio was found to be 136 (56.6%) for low digit ratio and 104 (43.4%) for high digit category. The low digit ratio category had 60% of males and 40% of females, whereas the high digit ratio category had 66% of females and 34% of males. The group was found to be very highly significant.

The very high risk group that formed around 212 (10.4%) of total population was further divided according to sex and digit ratio. The distribution of digit ratio was found to be 117 (55%) for low digit ratio and 95 (45%) for high digit ratio. The low digit ratio category had 61% of males and 39% of females, whereas the high digit ratio category had 57% of males and 43% of females. The group was found to be insignificant (Table 5).

The caries experience in both the dentitions was observed as 49% in very low risk category, followed by moderate risk (16.6%), high risk (12%), low risk (12%), and very high risk (10%). Low digit ratio was affected more as compared with high digit ratio in all the groups except in a very low risk group where the distribution was almost equal. Males were found to be more affected in low digit ratio groups and females were found to be more affected in high digit ratio group except the very high-risk category that was statistically insignificant. All the groups other than high-risk category were very highly significant and showed strong association with digit ratio.

**Table 5:** **Table 5:** The association of caries experience and digit ratio according to sex

*Caries exp*		*Digit ratio*		*Male (%)*		*Female (%)*		*Total (%)*		*Significance*	
Very low 997 (48.9%)		Low 497 (49.8%)		58		42		100		0.000	
		High 500 (50.2%)		48		52		100			
Low 249 (12%)		Low 136 (55%)		57		43		100		0.000	
		High 113 (45%)		44		56		100			
Moderate 339 (16.7%)		Low 226 (66.6%)		55		45		100		0.007	
		High 113 (33.4%)		47		53		100			
High 240 (12%)		Low 136 (56.6%)		60		40		100		0.000	
		High 104 (43.4%)		34		66		100			
Very high 212 (10.4%)		Low 117 (55%)		61		39		100		0.239	
		High 95 (45%)		57		43		100			

On analyzing the group it was seen that male population was prone to dental decay when the entire population was divided according to sex. It was also observed that the caries experience was found to be higher in primary dentition as compared with permanent dentition. Moreover, dft was found to be declining with age and DMFT was found to be increasing with age. This could be attributed to the change of primary dentition to permanent dentition (Table 5).

## DISCUSSION

Dental caries is one of the most prevalent infectious diseases to afflict mankind. The proportion of the world’s population affected by dental caries has increased dramatically as the refined carbohydrates has become available to the developed and the developing nations.^6^ The development of dental caries is dependent upon the critical interrelationship between susceptible host-tooth surface, specific oral bacteria, dietary carbohydrates, and the balance between the cariogenic and noncariogenic microbial population within saliva.^7^ It is also important to remember that one-fifth of the population accounts for about two-thirds of the total caries experience. Currently, it is difficult to identify the population who are at risk for developing dental caries with available screening methods. Therefore the present study attempts to identify an anatomical marker that works for an individual body that is also stable, reproducible, and constant throughout their life. This study also determines to find out if there is any interrelationship between digit ratio and caries experience in a given population of 2,037 children belonging to age group of 6 to 16 years.

The 2D:4D ratio has been much spoken and discussed in the medical sciences but as far as dentistry is concerned, it has not been explored much. Because of its early emergence, sexual dimorphism with respect to 2D:4D digit ratio is thought to be influenced by prenatal sex hormone regimes. In particular, high prenatal androgens may produce a low (masculine) digit ratio.^2,8^ These assumptions are partially supported by studies of males and females with congenital adrenal hyperplasia (CAH), a condition characterized by the overproduction of prenatal androgens.^9,10^

Manning et al^3^ and Brown et al^11,12^ found that digit ratio once formed remains stable throughout life. However, the length of finger will remain variable throughout the period of growth and development, but the ratio will remain more or less constant due to concentration of prenatal androgen exposure. Hence the present study was undertaken.

Total number of 2,037 Indian children who participated in this study were divided into three groups of digit ratio, i.e., low 2D:4D group (86%) followed by high 2D:4D (9%) group and equal 2D:4D (5%) group. The low 2D:4D group being the most dominant group comprises more than three-fourths of the total population. The mean digit ratio of high, low, and equal group was >1, 1, and <1, and the finding was in accordance with Manning et al,^3^ but in the present study we also found that this ratio has been seen in 86% of the Indian population. This was also in concurrence with Lakshmi et al,^13^ where a similar study was conducted on 500 children and found that 81% of the population fell into the low digit ratio category.

The low 2D:4D group being the most dominant group comprises more than three-fourths of the total population and was quite alarming. The raised values of the low digit ratio category prompted us to revisit the method used for the calibration of digit ratio, and it was found that the readings were given based on the mean values of ratio.^14^ The same criteria were applied in the present study by taking out the mean of both the values and finding the median value.

Fink et al^14^ conducted a study where he compared various studies done by several researchers and found that difference between low digit and high digit values were all below the value of 1.

Even in the present study, it was found that a strong occurrence of sexual dimorphism is existing but at different points, i.e., the mean ratio for men was found to 0.95 wherein for females it was 0.96 that was below the mean value of 1. Therefore, a new median value was calculated to define high and low digit ratio and the population was redivided into two categories, with the mean value being 0.95 and categorizing it as <0.95 and >0.95, according to South Indian population.

When the same population was divided according to gender to find out the sexual distribution, it was observed that low digit ratio group was dominated by males and high digit ratio was dominated by females, which is in accordance with George,^1^ Manning et al,^3^ and Fink et al^14^ studies.

In the low digit ratio, majority of the population was dominated by males but there was significant number of females also in that category that could be due to hormonal imbalances occurring in children, such as CAH.^10^ It was also found that in the present study significant number of women were falling into low digit ratio category. Large amount of luteinizing hormone (LH) and estrogen are found in individuals with low digit ratio category, and this may be attributed to the maternal testosterone that may cross the placenta and affect the differentiation of the ovaries and the digits. Thus, this could explain why females with low 2D:4D have low concentrations of estrogen and high level of testosterone.^3^ Apart from prenatal conditions, there might be other hormonal factors regulating the body, such as menstrual cycle, which is characterized by high fluctuating levels of different hormones, mainly estrogen and progesterone.^15^

Similarly in high digit ratio, majority of the population was dominated by females but there was a considerable number of males in that category that could be due to amount and the timing of exposure of prenatal testosterone and estrogen occurring in the womb. The developmental basis for this difference in digit ratio was well explained by Zheng and Cohn.^16^ Besides this, another possible reason could also be if the mother may expose the baby to more estrogen during the prenatal life, i.e., exposure to xenoestrogens.

Manning et al^3^ suggested positive correlation between 2D:4D ratios and levels of LH (right and left hands) and estrogen (right hand only). This finding led to the suggestion by Mayhew et al^15^ that 2D:4D ratio in the right hand may be more sensitive to hormone and was the reason that the digit ratio was recorded on the right hand of children.

The measurement of 2D:4D digit ratio was done by using a digital Vernier caliper. The measurement was made from the tip of the index/ring finger, i.e., by establishing the soft tissue contact without any pressure, till the midpoint of second basal crease of the digit. The measurements were repeated thrice to eliminate error. Two similar readings were considered. Similar methods were performed by Coyne et al^17^ and Manning et al^3^ and found the indirect methods to be unreliable. Hence in the present study, the direct method was used as examiner gets to see the subject in person for analyzing the subject fingers that is appropriate for the study.

The low digit ratio group was dominating in the nontaster group. This could be attributed to the reason that low digit ratio category is for testosterone-derived individuals who have organizational effects on the brain, and therefore studies indicate that individual with high testosterone levels has higher sugar requirements that runs in a loop, thereby sending feedback to brain for higher sugar consumption and running into a cause and effect mechanism.

When the caries was observed according to sex, males had higher caries experience compared with females.^18,19^ These findings was in accordance with Lin,^7^ where the study was conducted on 300 children and it was found that nontasters had higher caries experiences as compared with super tasters.

The primary dentition was found to be more affected, which may be due to morphology and enamel thickness. It was also noticed that as the child attains the permanent dentition, the mean value of DMFT had increased with age, suggestive of increased caries incidence at a later stage.

The direct relationship between digit ratio and dental caries in primary and permanent dentition suggested that the caries experience in primary dentition had a definite and positive correlation with the digit ratio. Therefore, digit ratio can be considered as a constant and stable anatomical marker to predict the risk of dental caries in primary dentition. Early diagnosis will not only give them healthy dentition and good oral health but will also go long way in preventing any sort of lifestyle disorders which they are likely to develop, thereby reducing the financial burdens on the family and health sector.

## CONCLUSION

It can also be said that digit ratio can act as a predictor that completely overrides the gender and ethnicity. The assessments and validations made in the present study were purely upon prenatal exposure and although there is the occurrence of sexual dimorphism, it cannot be totally relied upon sexual distribution.

## References

[1] George R (1930). Human finger types. Anat Rec.

[2] Putza DA, Gaulin SJ, Sporter RJ, McBurney DH (2004). Sex hormones and finger length. What does 2D:4D indicate?. Evol Hum Behav.

[3] Manning JT, Scutt D, Wilson J, Lewis-Jones DI (1998). The ratio of 2nd to 4th digit length: a predictor of sperm numbers and concentrations of testosterone, luteinizing hormone and oestrogen. Hum Reprod.

[4] Sacerdote C, Guarrera S, Smith GD, Grioni S, Krogh V, Masala G, Mattiello A, Palli D, Panico S, Tumino R (2007). Lactase persistence and bitter taste response: instrumental variables and Mendelian randomization in epidemiologic studies of dietary factors and cancer risk. Am J Epidemiol.

[5] (1997). World Health Organization. Oral health survey: basic methods.

[6] Vargas CM, Crall JJ, Schneider DA (1998). Socio-demographic distribution of pediatric dental caries: NHANES III, 1988-1994. J Am Dent Assoc.

[7] Lin BP (2003). Caries experience in children with various genetic sensitivity levels to the bitter taste of PROP. Pediatr Dent.

[8] Baileya AA, Hurd PL (2005). Finger length ratio (2D:4D) correlates with physical aggression in men but not in women. Biol Psychol.

[9] Tepper BJ (1998). Genetics of perception 6-n-propylthiouracil: a genetic marker for taste, with implications for food preference and dietary habits. Am J Hum Genet.

[10] McIntyre MH (2006). The use of digit ratios as markers for perinatal androgen action. Reprod Biol Endocrinol.

[11] Brown WM, Finn CJ, Breedlove SM (2002). Sexual dimorphism in digit-length ratios of laboratory mice. Anat Rec.

[12] Brown WM, Hines M, Fane BA, Breedlove SM (2002). Masculinised finger length patterns in human males with congenital adrenal hyperplasia. Horm Behav.

[13] Lakshmi CR, Radhika D, Prabhat M, Bhavana SM, Sai Madhavi N (2016). Association between genetic taste sensitivity, 2D:4D ratio, dental caries prevalence, and salivary flow rate in 6-14-year-old children: a cross-sectional study. J Dent Res Dent Clin Dent Prospects.

[14] Fink B, Neave N, Manning JT (2003). Second to fourth digit ratio, body mass index, waist-to-hip ratio, and waist-to-chest ratio: their relationships in heterosexual men and women. Ann Hum Biol.

[15] Mayhew TM, Gillam L, McDonald R, Ebling FJ (2007). Human 2D (index) and 4D (ring) digit lengths: their variation and relationships during the menstrual cycle. J Anat.

[16] Zheng Z, Cohn MJ (2011). Developmental basis of the sexually dimorphic digit ratio. Proc Natl Acad Sci USA.

[17] Coyne SM, Manning JT, Ringer L, Bailey L (2007). Directional asymmetry (right-left differences) in digit ratio (2D:4D) predict indirect aggression in women. Personal Individ Differ.

[18] Rupesh S, Nayak UA (2006). Genetic sensitivity to the bitter taste of 6-n propylthiouracil: a new risk determinant for dental caries in children. J Indian Soc Pedod Prev Dent.

[19] Verma P, Shetty V, Hegde AM (2006). Propylthiouracil (PROP) - a tool to determine taster status in relation to caries experience, Streptococcus mutans levels and dietary preferences in children. J Clin Pediatr Dent.

